# Chest X-ray Findings and Hyponatremia in COVID-19 Pneumonia Patients

**DOI:** 10.5339/qmj.2022.34

**Published:** 2022-08-05

**Authors:** Elmukhtar Habas, Abdussalam Abugrara Said, Ahmed Faidh Ramzee, Hafedh Ghazouani, Areen Fino, Mohamad A. Abu Khattab, Muna S. Al Masalamani, Arun Prabhakaran Nair

**Affiliations:** ^1^Internal Medicine Department, Hamad Medical Corporation (HMC), Doha, Qatar E-mail: faidhramzee@live.com; ^2^Department of Radiology, Al-Rumailah Hospital, Doha, Qatar; ^3^Department of Surgery, Hamad Medical Corporation (HMC), Doha, Qatar; ^4^Department of Statistics, Hamad Medical Corporation (HMC), Doha, Qatar; ^5^Department of Family Medicine, Hamad Medical Corporation (HMC), Doha, Qatar; ^6^Communicable Disease Center (CDC), Hamad Medical Corporation (HMC), Doha, Qatar

**Keywords:** Hyponatremia, COVID-19, pandemic, CXR findings, CXR findings score, CO-RADS

## Abstract

Background: The World Health Organization declared the coronavirus disease-2019 (COVID-19) a pandemic in December 2019. COVID-19 can affect most organs of the body but predominantly affects the lungs. Chest infection is associated with hyponatremia primarily due to inappropriate ectopic secretion of antidiuretic hormone. We conducted a six-month retrospective observational study to evaluate the relationship between chest X-ray (CXR) radiological findings and serum sodium levels. Our secondary goal was to assess the relationship between CXR findings and patient outcomes.

Aim of the Study: To assess the relationship between the initial CXR findings, hyponatremia severity, and outcome in COVID-19 infected patients.

Materials and methods: We conducted a retrospective review of CXR findings of COVID-19 patients aged > 18 years. The patients were healthy and had no history of hyponatremia before COVID-19 infection. All recruited patients were admitted to one of four hospitals in Qatar (Hazm Mebaireek General Hospital, Communicable Disease Center, and all affiliated quarantine centers managed under the Communicable Disease Centre, Mesaieed Hospital, and Ras Laffan Hospital) between March and June 2020. We excluded patients with factors that contributed to hyponatremia. Three score grades were established to describe the CXR findings. Patients were divided into three groups by the principal researcher according to their serum sodium levels. A radiologist evaluated the CXR findings with the patient and group information obscured. The principal researcher collected the X-ray scores for analysis with the serum sodium levels. We used SPSS for Windows, Version 15.0. (SPSS Inc., Chicago, IL, USA) and STATA Package Version 12.0 (StataCorp, College Station, TX, USA) to analyze the data. A *p*-value ≤  0.05 was considered significant.

Results: A total of 414 CXR patients with COVID-19 were recruited; 275 patients had hyponatremia and 139 had normal sodium levels and were used as the control group. Patients with normal serum sodium and hyponatremia were classified into three categories based on the CXR findings. Grade 0 (95), Grade 1 (43), and Grade 2 (137) hyponatremic patients were reported. The mean sodium levels were 133.6, 131.3, and 127.2 mmol/L for Grades 0, 1, and 2, respectively (*p* < 0.001). More than 95% of the patients who developed hyponatremia were >30 years. Moderate and severe hyponatremia was more prevalent in patients with Grade 1 or Grade 2 CXR findings and were >30 years.

Conclusion: Serum sodium levels in COVID-19 patients correlated well with the severity of the CXR findings observed at the early disease stage. Furthermore, simple CXR scores can be used to identify COVID-19 patients at a higher risk of hyponatremia, length of hospital stay, medical care support type, and mortality.

## Introduction

The coronavirus disease-2019 (COVID-19) pandemic is caused by severe acute respiratory syndrome due to Coronavirus 2 (SARS-CoV-2). SARS-CoV-2 has ravaged the world since December 2019, disrupting the global economy and healthcare systems. The number of worldwide COVID-19 cases reached 501,970,999 on April 14, 2022, of whom 6,190,360 died.^1^ Patients with COVID-19 may be asymptomatic, although mild upper respiratory tract infections or severe pneumonia can develop, leading to acute respiratory distress syndrome (ARDS), shock, and multiorgan failure.^2^ Extensive research has identified several risk factors that increase the susceptibility for developing severe illness; namely, older age, diabetes, chronic kidney disease, obesity, and chronic cardiovascular disease.^3^ There is great clinical benefit in establishing clinical and laboratory parameters that can help identify patients at risk of developing a long course of the disease, perform timely interventions, and identify novel therapeutic modalities to treat COVID-19 infection.

Recent studies have shown that the virus gains access to the host cells, particularly the lungs, via the angiotensin-converting enzyme-2 (ACE-2) receptor, introducing various degrees of tissue damage. It has been postulated that a low serum sodium level may lead to an upregulation of ACE-2 receptors, thereby inviting a more significant load of viral particles culminating in increased disease severity.^3^ Hyponatremia is one of the most common electrolyte abnormalities in patients with SARS-CoV-2 infection.^4,5^ Hyponatremia has been linked to an increase in the release of antidiuretic hormone (ADH) from hypothalamo-hypophyseal axis stimulation due to illness and stress, pneumonia-induced lung injury, direct/indirect renal injury, or a cytokine storm. Increased ADH secretion causes a syndrome of inappropriate antidiuretic hormone secretion (SIADH).^5,6^

Chest X-ray (CXR) is not a sensitive method to detect lung changes in viral pneumonia at its early stages, whereas chest computed tomography (CT) imaging has been proven to be a reliable diagnostic tool for assessing lung involvement and severity during the early stages of a COVID-19 infection.^7^ CT findings have a sensitivity of up to 90% and have been promoted as the first-line imaging modality for diagnosis in different countries.^7^ Nevertheless, CXR may have a role in diagnosing and evaluating the progression of lung involvement, particularly in the intensive care setting.^7^ A chest CT scan standardized scoring system, known as the COVID-19 Reporting and Data System (CO-RADS), is currently available and widely used.^8,9^ Although experimental CXR scoring systems have been proposed, none have been validated for a SARS-CoV-2 chest infection.^10^ Hui et al. concluded that the degree of CXR changes correlates more with the severity of SARS-CoV-2 chest infection than with laboratory markers.^10^ The same study showed that normal or very mild changes are associated with a more stable disease course, although CXRs were done 6–10 days after symptom onset. Scoring systems for ARDS, such as the radiographic assessment of lung edema (RALE) score are well known and have been used to evaluate the severity of pneumonia and the risk of intensive care unit (ICU) admission.^11–13^ Wong et al. used the RALE score to assess the time course and severity of CXR changes in patients with SARS-CoV-2 and reported significant bilateral lower zone consolidation 10–12 days after symptom onset with a sensitivity of 69%.^14^ Another scoring system described by Taylor et al. is used to assess the degree of severe acute respiratory infection by non-radiologists.^15^ This score has five categories according to the severity of the CXR findings: 1 – normal; 2 – patchy atelectasis and/or hyperinflation and/or bronchial wall thickening; 3 – focal consolidation; 4 – multifocal consolidation, and 5 – diffuse alveolar changes. Only one study has assessed this scoring system in patients with SARS-CoV-2 infection; however, the correlation between severity and the radiographic findings was poor.^16^ Borghesi et al. recently described a scoring system (the Brixia score) for SARS-CoV-2 infection based on dividing the CXR into six zones, scoring each zone based on the presence of infiltrates and combining the scores into one definitive score ranging from 0 to 18.^17^ They showed that a higher score was associated with increased mortality; however, this score must be validated by other studies. CXR is more readily available than CT; it is a cost-effective tool when used appropriately, particularly in overburdened healthcare systems. This study correlated the earliest CXR findings in both lungs of COVID-19 pneumonia patients with serum sodium levels and their likely outcomes.

## Materials And Methods

Ethics Committee approval was obtained from the Medical Research Centre (MRC) (Number: MRC-05-211) for this retrospective observational study. COVID-19 pandemic management in Qatar is coordinated by the Hamad Medical Corporation, which is under the purview of the Ministry of Public Health. At the beginning of the pandemic, four main hospitals were designated as COVID-19 treatment facilities (Hazm Mebaireek General Hospital, the Communicable Disease Center, and all affiliated quarantine centers managed under the Communicable Disease Center, Mesaieed Hospital, and Ras Laffan Hospital). We reviewed the clinical and imaging records of all COVID-19 pneumonia patients aged >18 years with polymerase chain reaction-confirmed SARS-CoV-2 infection who were admitted to any of these facilities between March and June 2020. The exclusion criteria eliminated any other contributing factors for hyponatremia, including chronic SIADH, diarrhea, hyperlipidemia, chronic low serum sodium, chronic kidney disease, an endocrine disorder, diabetes, hypertension, cardiovascular disease, and other known diseases that may cause hyponatremia or affect the outcome of COVID-19 infection.

**Figure 1.**
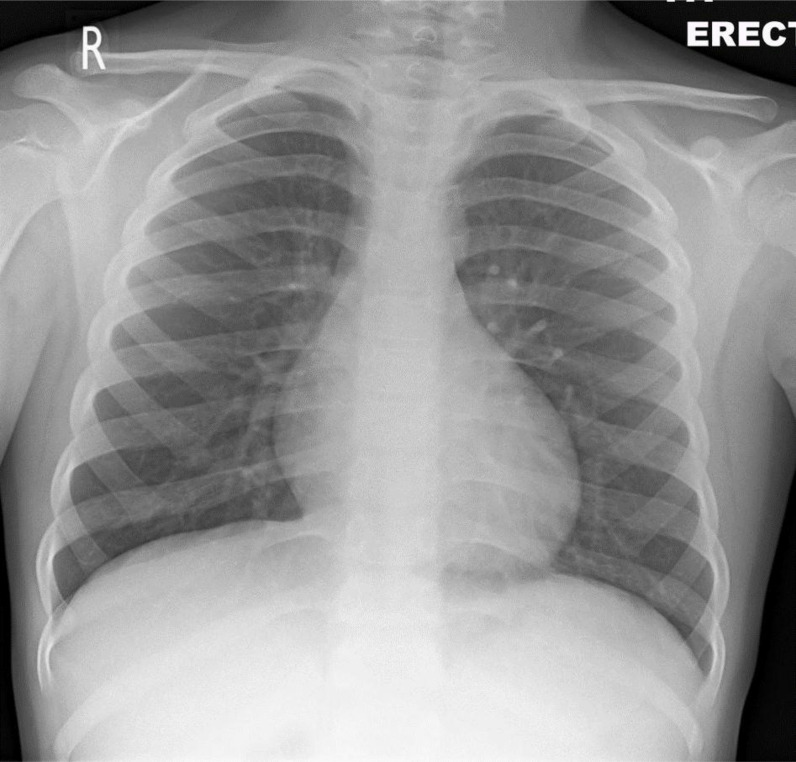
Grade 0 chest X-ray: no abnormal radiological findings

**Figure 2.**
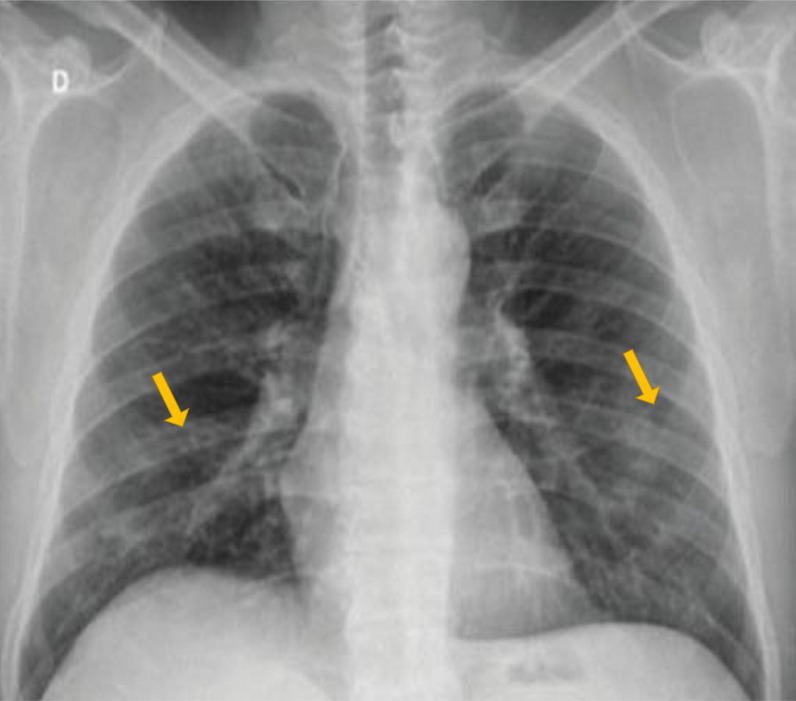
Grade 1 chest X-ray: Alveolar consolidation either unilaterally or bilaterally. Example shows bilateral peripheral alveolar consolidation in the lower lobes.

**Figure 3.**
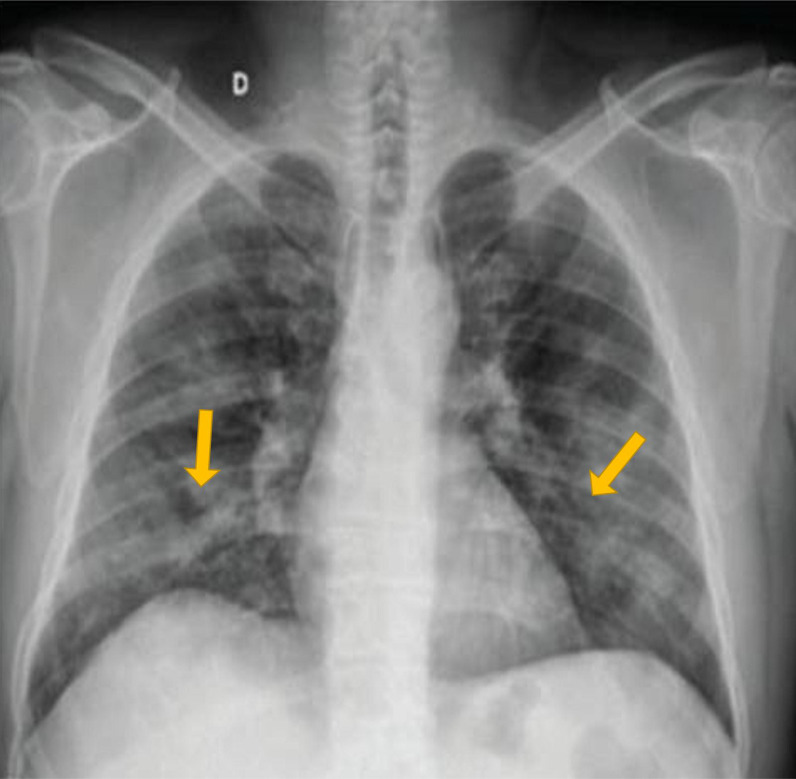
Grade 2 chest X-ray: bilateral alveolar consolidation affecting the whole lung lobe (pan-lobar consolidation) or widespread severe findings.

**Figure 4.**
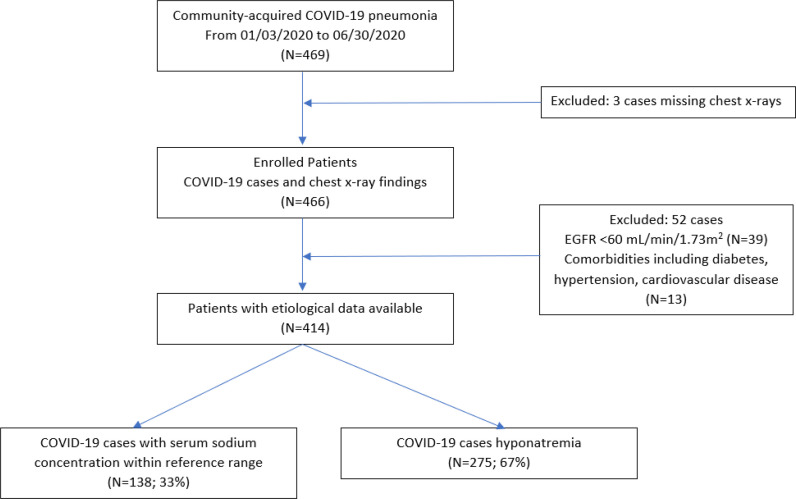
Flow chart of the patients selected for the study

The hyponatremic patients were divided into three subgroups according to their serum sodium levels. The severity of the hyponatremia was classified according to the Joint European guidelines.^18^ These guidelines proposed hyponatremia ranges of low (130–135 mmol/L), moderate (125–129 mmol/L), and severe ( < 125 mmol/L).^18^ The CXR findings used to describe the grades were discussed thoroughly among the team members, including the radiology consultant. A simple scoring system was proposed to describe the initial CXR findings. The CXR findings were scored as follows: Grade 0 indicated the CXR had unremarkable findings (i.e., no significant abnormalities on the CXR at the hilum or peripheral lung fields) (Figure 1); Grade 1 indicated the presence of either mild unilateral or bilateral peripheral alveolar consolidation at the lower lobes of lungs (Figure 2), and Grade 2 indicated bilateral alveolar consolidation affecting the whole lung lobe (pan-lobar consolidation) or widespread severe findings (Figure 3). Patient and group identifying information was hidden from the entire team (except the principal investigator), including the radiologist who reported the CXR observations. Serum sodium and X-ray scores were arranged in a Microsoft Excel spreadsheet (Microsoft Corp., Redmond, WA, USA). A statistician processed and organized the data into three subgroups of eunatremic and hyponatremic levels according to the CXR score grade. The demographic characteristics, complete blood count parameters, and serum potassium levels were included in the statistical analysis to assess any interactive effects on patient outcomes and the CXR grades. The biochemical variables included were those taken at the time of the CXR or the nearest to the time of the CXR. The process of case selection is outlined in Figure 4.

### Statistical analysis

We used SPSS for Windows, version 15.0. (SPSS Inc., Chicago, IL, USA) and STATA Package version 12.0 (StataCorp, College Station, TX, USA) for the exploratory data analysis and descriptive statistics, respectively. The Mann-Whitney *U*-test or *t-*test was performed for two groups according to the distribution or homogeneity of the continuous variables. Categorical variables are reported as frequencies and percentages. Nominal and categorical variables were compared using the chi-square test and Fisher's exact test, respectively. The association between serum sodium levels and outcomes was assessed by univariate analysis. Logistic regression analysis with stepwise backward elimination was performed to confirm the risk factors of hyponatremia at diagnosis in patients with COVID-19. The means of the parameters between the three CXR groups were compared using analysis of variance (ANOVA) for normally distributed variables and the Kruskal-Wallis test for non-normally distributed variables. Missing data were handled primarily by complete case analysis at the time of the statistical analysis. As a secondary analysis, missing data were imputed using multiple imputations. A *p*-value ≤ 0.05 was considered significant.

## Results

In total, 469 admitted patients with COVID-19 pneumonia were identified in the four qualifying hospitals in Qatar between 1 March 2020 and 30 June 2020. Three patients were excluded due to the lack of X-ray images, and 52 patients were excluded due to the aforementioned hyponatremia contributing factors; thus, 414 patients were enrolled in this study (Figure 4). Most patients were male (n = 255), and only 20 were female. A total of 275 patients had hyponatremia (Table 1) and 139 proven COVID-19 patients had normal serum sodium levels throughout their COVID-19 course. The eunatremic patients served as the control group and were divided into three groups according to their CXR scores. The control group contained 139 patients; 120 patients with Grade 0, 16 with Grade 1 and 3 patients with Grade 2 CXR findings. In the hyponatremia group, 95 patients had Grade 0, 137 had Grade 2, and 43 patients had Grade 1 CXR findings (Figure 2).

**Figure 5.**
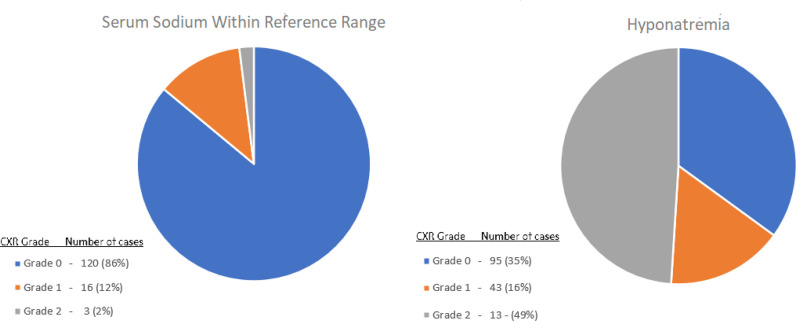
Distribution of the hyponatremic and eunatremic patients according to the X-ray grade.

**Figure 6.**
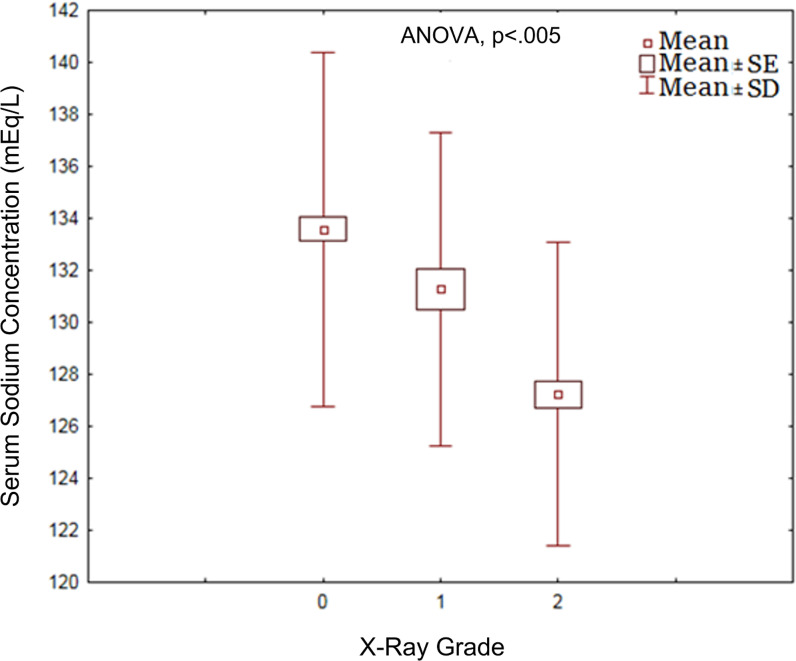
Box plot presenting the difference in mean serum sodium levels between X-ray grades among patients with COVID-19.

The statistical analysis revealed a negative correlation between the X-ray grade and serum sodium concentration (r = − 0.115, *p* < 0.001). The mean sodium level in Grade 0 patients was 133.6 ± 6.8 mmol/L, 131.3 ± 6 mmol/L in Grade 1, and 127.2 ± 5.8 mmol/L in Grade 2 (*p* = 0.000; Table 2, Figure 5). The box plot in Figure 6 displays the difference in mean serum sodium levels between the X-ray grades. Fewer than 5% of patients who developed hyponatremia were < 30 years of age. Moderate and severe hyponatremia with worse CXR grades (Grade 1 or 2) were observed in patients >30 years. However, Grade 0 CXR findings were reported in 86% of COVID-19 pneumonia patients with a serum sodium concentration within the reference range, and only three patients were scored with Grade 2 CXR findings. Surprisingly, the platelet count decreased significantly and directly corresponded to the grade of the CXR findings in hyponatremic patients (*p* = 0.02). Unexpectedly, serum potassium, creatinine, hemoglobin (Hgb), and white blood cell (WBC) levels were within the normal ranges in hyponatremic patients. A multivariate logistic regression adjusted for age, serum creatinine, serum potassium, WBCs, hemoglobin, and platelet count was conducted to assess their effect on the relationship between the CXR grade and serum sodium. The changes in the platelet count were significantly associated with the CXR findings and serum sodium levels stratified by illness severity among patients with SARS-CoV-2 infection.

The correlation of determination and the *p* values are shown in Table 3. Multiple logistic regression analysis demonstrated that the relationship between gender and the platelet count remained an independent, significant risk factor associated with CXR severity and the serum sodium level stratified by illness severity among patients with SARS-CoV-2 infection (Table 4).

ANOVA and the difference between the three groups according to Tukey's multiple comparison test revealed that the mean age of the eunatremic patients was 36–67 years. The mean age of the hyponatremic patients was 45–50 years, with a significant difference between the two groups (*p* < 0.05; Table 5). Comparisons of sex in the X-ray grades of the same group and between the two groups were not significantly different. The serum potassium concentration was toward the lower side of the reference range in the hyponatremic and eunatremic patients; however, ANOVA revealed a significant difference in the mean serum potassium changes between the two groups (*p* = 0.03). The WBC count was within the normal range in the two groups; however, it tended to be higher in the hyponatremic patients who had Grade 1 and Grade 2 X-ray findings (*p* = 0.61; Table 5). The thrombocyte count was within the normal range in both groups, although it tended to decrease. The decrease in the platelet count was more profound in hyponatremic patients with Grade 1 CXR findings (198.53 ± 62.78 cells/μL) than in the control group patients. The difference in the thrombocyte count between the two groups was significant (*p* = 0.002) (Table 5).

Fifteen patients (8%) died in the hyponatremia group: two had Grade 1 CXR findings and 13 patients had Grade 2 CXR findings. Three of the 15 patients in the medical ward died unexpectedly, and only one patient of the three received ventilator support during resuscitation. The other 12 patients were admitted to the ICU. Eleven patients developed severe desaturation and required mechanical ventilatory support, whereas the other died without ventilator support. The average lengths of stay were 17 and 29 days in the medical ward and ICU, respectively. The cause of death was septic shock in nine patients, and seven of these nine patients developed ARDS. The other six patients died possibly due to pulmonary embolism and acute kidney injury or cardiac complications, such as carditis or arrhythmias.

## Discussion

Interestingly, hyponatremia was reported as one of the most common electrolyte abnormalities during the SARS pandemic in 2003.^19^ Nearly 60% of patients reported low sodium levels in a study from Hong Kong.^19^ Similarly, hyponatremia is one of the most common electrolyte abnormalities in patients with SARS-CoV-2 infection.^4,5^ The pathogenesis of hyponatremia in patients with COVID-19 infection is uncertain; however, low serum sodium during a COVID-19 infection seems to be primarily due to an inappropriate increase in ADH secretion. Higher ADH secretion appears to occur because of abnormalities in the hypothalamo-hypophyseal axis. These abnormalities could be due to inflammation of these glands by the SARS-CoV-2 infection.^20^ Contributory factors, such as disease stress, inflammatory lung injury, and a cytokine storm can contribute to hyponatremia.^5,6^ Another possible mechanism of low serum sodium in patients with COVID-19 is linked to the deposition of virus particles in the glomeruli and tubules, which directly damage these structures.^21^ Furthermore, COVID-19 infection causes acute proximal tubular injury associated with glomerular thrombi due to direct glomeruli endothelial damage.^22^ These abnormalities in the nephrons may cause a defect in sodium handling, resulting in hyponatremia.

Pneumonia is a well known cause of SIADH and could be an inciting factor for hyponatremia in patients infected with SARS-CoV-2.^23,24^ However, other mechanisms of hyponatremia are being investigated.

Direct renal injury by SARS-CoV-2 has occurred in the proximal convoluted tubules via viral particles.^25^ This process is mediated via the ACE-2 receptor, which facilitates the entry of viral particles into cells, resulting in damage,^3,25,26^ acute kidney injury, and electrolyte abnormalities. These receptors are present in the lungs, heart, muscle, bowel, and kidneys, which explains the increased susceptibility of those organs to SARS-CoV-2-related injury.^27^ This process is compounded by a cytokine storm, with increased levels of interleukin (IL)-6, which cause glomerular damage and tubular necrosis.^28^

The present study results reveal a significant association between COVID-19 pneumonic CXR findings and serum sodium concentration. According to the CXR score used in the present study, infection severity was negatively correlated with the sodium level (i.e., the higher the X-ray grade, the lower the serum sodium concentration).

CXR radiological assessments play an essential role in diagnosing and predicting the prognosis in patients with SARS-CoV-2 infection; hence, chest CT is useful as a diagnostic tool in some countries.^13^ CO-RADS is an established grading system for COVID-19 pneumonia based on CXR findings.^29^ However, given the high cost and limited availability of CT machines even at leading hospitals, and the large influx of COVID-19 patients, CT was not an appropriate tool for wide use during the pandemic in new hospitals that were not well equipped. However, CXR machines are widely available in most centers and are relatively more accessible, and X-rays are less expensive and easier to perform than CT scans. Unfortunately, X-rays have no validated scoring system (such as CO-RADS for CT scans), making X-ray findings alone a less objective tool to assess COVID-19 severity, particularly during the early stage of COVID-19 pneumonia.^30^ Several scores have been described for ARDS, such as the score described by Borghesi et al. for SARS-CoV-2 infection (i.e., the Brixia score).^17^ This score divides the CXR into six zones, and each zone is scored based on the presence of infiltrates; the combined score ranges from 0 to 18.^17^ CXR findings in our study were classified according to the severity and the extension of CXR observations into three grades. We detected a negative correlation between the grade of the CXR findings and serum sodium level (Table 2). Therefore, the CXR score introduced in this study was an objective scoring method for serum sodium that can be used to predict the severity of hyponatremia. Furthermore, the degree and onset of hyponatremia are important when predicting mortality or morbidity.^31^ Therefore, early diagnosis and treatment of hyponatremia are essential to improve morbidity and mortality; consequently, the CXR score helped improve the morbidity and mortality rates of COVID-19 patients.

Hematological abnormalities are not uncommon in COVID-19 patients. Surprisingly, the platelet count was significantly low during COVID-19 infection, and the decrease was greater for the Grade 2 CXR patients in the present study. Although the lymphocyte and platelet counts decreased without a significant change in the WBC value, the thrombocyte count usually does not decrease to a level that causes significant bleeding.^32^ There is no apparent explanation for the underlying mechanisms of thrombocytopenia; however, three theories have been postulated. The first theory is based on the direct effect of virus infection on bone marrow cells, which suppresses platelet production. Platelet progenitor cells are indirectly damaged during a cytokine storm, which halts platelet production. Moreover, lung changes that occur with the infection indirectly decrease platelet synthesis.^33^ The second possible mechanism is immune system activation by forming autoantibodies as in human immunodeficiency virus-infected patients, which increases the consumption of platelets.^34^ Third, disseminated intravascular coagulation precipitates platelet aggregation in lung vessels, which increases platelet consumption.^33^

A direct relationship has been reported between the severity of COVID-19 pneumonia and IL-6 serum levels.^8^ IL-6 is superior to C-reactive protein and other inflammatory markers for predicting respiratory failure in COVID-19 patients.^35^ Furthermore, IL-6 seems to be an essential factor that causes immune dysregulation and ARDS in patients with COVID-19.^36,37^?twb> Interleukin inhibitors (e.g., tocilizumab) improve hyponatremia and reduce the risk of death.^9^ Despite that IL-6 was not measured (a limitation of retrospective studies), it was likely very high and may have played a role in developing hyponatremia and pneumonia severity at least in some of our patients.

In an analysis of five studies with nearly 1,500 patients, Lippi et al. reported that severe COVID-19 is associated with lower serum sodium, potassium, and calcium levels.^38^ Low serum potassium in COVID-19 patients was also reported by Alfano et al.^39^ We found a significant association between the severity of COVID-19 and potassium levels, as nearly 86% of patients had serum potassium levels within the lowest normal of the reference range; however, this did not correlate significantly with pneumonia grade. Hypokalemia is a common electrolyte disturbance in patients with COVID-19, particularly in patients undergoing diuretic therapy.^39^ In the present study, serum potassium was at the lower end of the normal range, particularly on Grade 0 CXR findings although there was no statistical significance (Table 1). The normal serum potassium level reported in our patients can be explained due to the recommendation to replace potassium when its level is ≤ 3.1 mmol/L.

### Limitations

This was a retrospective study conducted at the onset of the pandemic in Qatar (March–June 2020). Given the nature of the study, we could not validate the scoring system with the patients’ clinical condition at that time or with the CT scoring system because most of our patients had not had a chest CT. The study would have benefited from patients having had chest CT scans at the same time as the X-ray, which would allow a comparison between our scoring system and CO-RADS. The CXR used in this study was the first CXR done at presentation, unlike other studies that used a CXR performed later (6–10 days after presentation), making our scoring system validation inappropriate according to previous scoring systems. The use of the first CXR, which was done at presentation, may explain the minor CXR findings reported in this study. However, a significant correlation was observed between hyponatremia, the hematological changes, and outcome. Serum IL and vasopressin hormone levels were not measured because it was a retrospective study. Nevertheless, an association between the severity of hyponatremia, pneumonia, outcomes, and the CXR findings was detected. The other limitation is the higher number of male patients, which may be due to the male predominance in the Qatar population (Male: female ratio = 2,043,351: 782,935 as of March 2022).^40^

## Conclusion

Serum sodium concentration was strongly correlated with the severity of CXR findings during the early stages of COVID-19 pneumonia. Although the CXR score used in this study was not validated, it appears to be a reasonable scoring method to predict hyponatremia severity, hospital stay, level of medical support required, and mortality in patients with COVID-19 pneumonia. However, further research is required to validate this scoring system and compare it with CT-CORAD and other scoring systems. Therefore, we urge clinicians and researchers to try the CXR scoring system described in this study, particularly in low-income countries, because CXR is less expensive, more widely available, and faster to perform than a CT scan.

### Authors’ Contributions

Habas E and Ahmed FR initiated the study by writing the approval protocol. These two authors were the principal manuscript editors, sharing equally. Said A graded the X-rays and reported all X-rays findings as grades. Ghazouani H performed all statistical analyses and generated the tables and figures. The other authors helped with data collection, processing, and revising the manuscript.

Conflict of Interest: None of the authors has a conflict of interest to declare.

Financial Support: HMC Research Center financially supported the publication fees for the study.

## Figures and Tables

**Table 1:** Group statistics comparing the COVID-19 patient demographic pattern and laboratory data by hyponatremia category and X-ray grade

	Hyponatremia (n = 275)			

Parameter^a^	X-ray Grade 0 (n = 95)	X-ray Grade 1 (n = 43)	X-ray Grade 2 (n = 137)	*p*-value

Age (years)	45.7 ± 13.5	46.8 ± 12.6	50.4 ± 11.0	0.512

Male	85 (89%)	42 (98%)	128 (94%)	0.206

Female	10(11%)	1(2%)	9(6%)	

WBC count (cells/uL)	7.62 ± 3.01	7.63 ± 4.50	7.28 ± 3.55	0.720

Creatinine level (umol/L)	80.25 ± 18.45	78.85 ± 18.50	84.22 ± 20.21	0.162

Potassium level (mmol/L)	3.93 ± 0.39	3.95 ± 0.51	4.00 ± 0.56	0.541

Hgb (g/dl)	14.15 ± 1.74	14.04 ± 1.52	13.69 ± 1.78	0.127

Platelet count (cells/uL)	234.48 ± 70.42	198.53 ± 62.78c	214.87 ± 76.06	0.02


^a^Data are presented as mean ± SD or frequency (%)

WBC- White blood cells, Hgb- Hemoglobin

**Table 2:** Association between the CXR findings and the serum sodium level stratified by illness severity

X-ray grade	N	%	Serum sodium concentration		Confidence		The *p*-value for each group^a^	The *p*-value across the 3 groups^b^

			Mean	Std.Dev.	-95%	95%		

0	95	52.18%	133.6	6.8	132.7	134.5	0.000	0.000

1	43	14.32%	131.3	6	129.7	132.9	0.000	

2	137	33.50%	127.2	5.8	126.2	128.2	0.000	


Data are presented as mean ± SD or frequency (%).

Analysis of variance (ANOVA) and the Bonferroni post hoc test were utilized to examine the difference in the serum levels of the variables between the X-ray grade radiological pneumonia group findings (0 – no involvement and no lung abnormalities);

1 – Less than 25–50% (interstitial infiltrates or interstitial and alveolar infiltrates [interstitial predominance]); 2–50% to >75% involvement (interstitial and alveolar infiltrates [alveolar predominance])

^a^ *p*-value for each group (Bonferroni post hoc test)

^b^ *p*-value across the three groups (ANOVA)

**Table 3:** Multivariate regression analysis of factors associated with SARS-COV-2 infection by hyponatremia category and X-ray grade.

	Coef	SE Coef

Constant	0.631	0.63

Age	-0.0004	0.003

WBC (cells/uL)	0.0027	0.0129

Creatinine (umol/L)	0.001	0.0025

Potassium (mmol/L)	0.0931	0.095

Hgb (g/dl)	-0.016	0.0294

Platelet (cells/uL)	-0.002	0.0000


WBC- White blood cells, Hgb- Hemoglobin

**Table 4:** Pearson's and Spearman's correlation analyses between the X-ray grade measurements and the laboratory data.

Parameter	r	p-value	Relationship Strength of Variables

Age (years)	0.134	< 0.05	Small

Male	0.014	0.831	Trivial

Female	0.004	0.912	Trivial

WBC count (cells/uL)	0.106	0.122	Small

Creatinine level (umol/L)	0.077	0.294	Trivial

Potassium level (mmol/L)	0.047	0.487	Trivial

Hgb (g/dl)	0.106	0.121	Small

Platelet count (cells/uL)	0.104	< 0.05	Small


WBC- White blood cells, Hgb- Hemoglobin

**Table 5:** Means of the baseline measurements by the serum sodium concentration level and chest X-ray grade among patients with COVID-19.

Parameter	Hyponatremia (275) patients			Normo-natremia (139) patients			*p*-value

	X-ray Grade 0 (n = 95)	X-ray Grade 1 (n = 43)	X-ray Grade 2 (n = 137)	X-ray Grade 0 (n = 120)	X-ray Grade 1 (n = 16)	X-ray Grade 2 (n = 3)	

Age (years)	45.7 ± 13.5	46.8 ± 12.6	50.4 ± 11.0	36.0 ± 11.6	43.5 ± 13.5	67 ± 11.1	0

Male	85 (89%)	42 (98%)	128 (94%)	114 (93%)	14 (87%)	2 (67%)	0.61

Female	10(11%)	1(2%)	9(6%)	6(7%)	2(13%)	1(33%)	0.124

WBC count (cells/uL)	7.62 ± 3.01	7.63 ± 4.50	7.28 ± 3.55	7.10 ± 2.13	6.46 ± 0.99	6.37 ± 2.85	0.61

Creatinine level (umol/L)	80.25 ± 18.45	78.85 ± 18.50	84.22 ± 20.21	79.17 ± 11.63	93.41 ± 9.98	115 ± 17.4	0.43

Potassium level (mmol/L)	3.93 ± 0.39	3.95 ± 0.51	4.00 ± 0.56	4.17 ± 0.37	4.12 ± 0.33	3.3 ± 0.35	0.03

Hgb (g/dl)	14.15 ± 1.74	14.04 ± 1.52	13.69 ± 1.78	14.65 ± 1.41	13.61 ± 1.12	12.81 ± 0.89	0.14

Platelet count (cells/uL)	234.48 ± 70.42	198.53 ± 62.78	214.87 ± 76.06	260.77 ± 64.24	228.23 ± 42.36	201.56 ± 46.12	0.002


Data are presented as mean ± SD or frequency (%)

Analyzed using ANOVA and Tukey's multiple comparison, Friedman's test, or Dunn's post hoc test for the differences between the three groups
